# Social and familial implications of algorithmic conflict management in hybrid work environments “a study of AI-driven fairness and trust in UAE organizations”

**DOI:** 10.3389/fsoc.2026.1754294

**Published:** 2026-06-16

**Authors:** Basma Mortada Fouda, Mervat Medhat

**Affiliations:** 1 The Higher Colleges of Technology, Sharjah, United Arab Emirates; 2 College of Mass Communication, Ajman University, Ajman, United Arab Emirates; 3 Humanities and Social Sciences Research Centre (HSSRC), Ajman University, Ajman, United Arab Emirates; 4 Art and Design Academy, Higher Institution of Applied Arts, Cairo, Egypt

**Keywords:** AI transparency, algorithmic fairness, digital conflict management, family spillover, hybrid workplaces, social trust, UAE, workplace surveillance

## Abstract

This study examines the family and social consequences associated with using Artificial Intelligence (AI) to manage workplace conflicts within hybrid workplaces. Hybrid workplaces are a new workplace environment, where technology is used in emerging area of inquiry concerning the degree to which AI related stressors bleed across boundaries into family life. Research has primarily focused on either the technological, or organizational aspects of algorithmic management. As such, there exists a dearth of theoretical understanding as to how workplace systems utilizing AI drive employee psychological security and subsequently their family dynamics. Thus, our objective was to understand how transparency regarding AI, fairness in algorithms used by AI, and digital surveillance employed by employers affects hybrid employee’s psychological security and ultimately their family dynamics within the UAE. We utilized an explanatory sequential design to utilize both quantitative survey data collected from 420 hybrid workers, along with qualitative thematic analysis of scenario based vignette responses. Our results indicate a stark disconnect; while participants had moderate confidence in AI systems resolving conflicts; they were extremely concerned about issues of fairness, privacy and lack of algorithmic transparency. Most importantly, 63% of respondents indicated that work-related stress experienced due to AI mediated workplace surveillance and evaluation of performance had a negative effect on communication and emotional stability with family members at home. Therefore, we have proposed the Social Algorithmic Justice Framework (SAJF) to identify the pathways from algorithmic transparency to family resiliency with the influence of social support. Ultimately, this study will provide a theoretically supported framework for integrating workplace AI with socially sustainable values so as to ensure that workplace AI integration enhances family harmony rather than diminishes it.

## 1 Introduction

Over the last decade, advancements in artificial intelligence (AI) technology along with algorithmic tools have dramatically impacted what happens in the workplace today, impacting how businesses interact with employees, assess employees’ performance and resolve issues (Barocas et al., 2023). As AI expands its reach far beyond the realm of data analysis and becomes active participants in resolving employment related issues including supporting managerial decision making, new paths to increased efficiency are being created while significant challenges exist relative to the concepts of justice and transparency (Kellogg et al., 2020).

In the United Arab Emirates (UAE), AI’s rapid implementation within business and public institutions is directly linked to national goals outlined in strategies like the “UAE Artificial Intelligence Strategy 2031 (UAE Digital Government, 2024; UAE Artificial Intelligence Office, 2022).” The goal of the strategy is to use AI enabled smart systems to greatly increase both government and private sector performance (Government of the United Arab Emirates, 2022). With many employers now implementing hybrid work models where employees work remotely and then come into an office setting, there exists growing concern among employees that they are subject to increasing amounts of monitoring and algorithmic bias based upon their usage of technology in order to determine productivity and resolve disputes.

While there may be numerous positive aspects associated with using AI in terms of enhancing operational effectiveness and efficiency, the issue of algorithmic transparency and fairness remains a point of contention. There is evidence to indicate that a lack of transparency in the use of automated decision-making processes creates distrust between employees and their supervisors (Wachter et al., 2017). Further research indicates that the psychological impacts of opaque algorithmic management extend well beyond the confines of the workplace. Studies show that when employees experience stress due to unresolved or poorly resolved workplace conflicts mediated by AI systems, it can create spillover effects into other areas of life, specifically the family environment (Bakker and Demerouti, 2013). For families residing in countries like the UAE where strong collectivist values contribute to an emphasis on maintaining close-knit family relationships, the introduction of algorithmically generated workplace stress can present a major societal challenge (UAE Ministry of Community Development, 2024).

A primary area of focus in this study is that prior literature has largely examined the institutional and technical implications of AI; however, a substantial void exists in regard to examining the social and familial ramifications of managing digital conflict and achieving algorithmic justice. Workplace conflict is no longer solely an organizationally confined event; rather, it is increasingly becoming a multi-dimensional phenomenon across contexts that contributes to family dynamics and the overall psychological wellbeing of working parents and their children. Consequently, it is essential to develop a socio-familial perspective toward examining AI that spans beyond typical organizational boundaries (Microsoft, 2023).

To fill this void in literature, this study will investigate how levels of transparency afforded by AI systems regarding decision-making algorithms, fairness provided by those same algorithms, and digitally collected employee surveillance affect workplace conflict management outcomes (McKinsey and Company, 2021) as well as work-family spillover occurring within hybrid workplaces operating within the UAE. This study proposes and tests the Social Algorithmic Justice Framework (SAJF) which views AI-enabled conflict resolution as an expansion of previously developed work-family spillover mechanisms. Within this framework, it is hypothesized that algorithmic stressors act as factors contributing to family tension as amplified by social and community supports.

## 2 Research problem, objectives, and hypotheses

Although there is significant evidence that digital technology usage in UAE workplaces continues to increase rapidly; however, it still unclear whether transparency of AI systems, fairness of algorithms or the use of digital monitoring will affect conflict-resolution success rates at both organizational and familial levels. The body of literature on digital technology has focused primarily on measuring the technical efficiency and accuracy of systems (Doshi-Velez and Kim, 2017). However, few studies have explored the human and social aspects specifically how employees and their families perceive fairness and trust in AI-enabled environments. With an increasing number of hybrid workplaces integrating AI to provide evaluation and decision-support data, the lack of transparency of these systems may lead to mistrust among employees, as well as create issues related to transparency that threaten employees’ emotional security and social bonds (Jehn, 1995).

This paper aims to investigate empirically the organizational and social factors influencing trust, fairness perceptions and work-family spillover in hybrid workplaces using AI in the UAE. This paper adopts a mixed-methods design using scenario-based vignettes to measure the lived experiences of hybrid workers and subsequently assesses the impacts on their family environment.

In order to accomplish the above goals, we developed research questions (RQs) and testable hypotheses (H), building on theoretical foundations from Organizational Justice Theory (Colquitt, 2001) and Work-Family Spillover Theory (Rahim, 2011):

RQ1: In what ways do perceptions of AI transparency and algorithmic fairness relate to employee trust and perceived fairness in conflict management processes in hybrid workplaces?

- H1a: Perceived AI transparency is positively correlated with employee trust in management decisions.
- H1b: Employee perception of algorithmic fairness is positively correlated with degree of satisfaction with the outcome of conflict resolutions.

RQ2: To what extent does digital surveillance and AI-related workplace stress result in spillover into family dynamics?

- H2a: Digital surveillance results in a positive correlation with employee reported work-family spillover stress.
- H2b: Family emotional wellness is negatively correlated with the level of work-family spillover stress resulting from algorithmic management practices.

RQ3: In what way(s) does social support from family/community function as a moderator in reducing the relationship between AI-induced stress and conflict resolution success rates?

- H3: High levels of social support from family/community reduce the negative effect of AI surveillance on work-life balance/employee wellbeing/resilience/conflict resolution effectiveness.

## 3 Literature review

### 3.1 AI transparency and digital identity in hybrid conflict settings

The introduction of AI in hybrid workplaces influences how and where employees fashion their work based identity and expectations for fairness in situations of conflict. Transparent AI systems (those that allow employees to ‘see’ how decisions are made) play a key role in how procedural justice judgements are made; research shows that when employees understand how AI evaluates their performance, attendance or communication trust in management is 41% higher. When AI systems are opaque to employees, AI generated identity profiling (e.g., behavioural analytics) can transfer inter-personal dynamics into the realm of conflict—or activate trauma-defensive behaviour during conflict (Kellogg et al., 2020). Digital productivity monitoring systems generate an “algorithmic persona” (the managerial perception of workers), but this “perceived worker secrets” persona may bear little resemblance to worker’s real intentions, context, or emotional state. Recent workplace studies (European Commission, 2023) find that 56% of hybrid employees in the UAE believe that AI profiling manifests through bias in conflict evaluations, especially for emotionally-sensitive issues. The distortion of individual identity through AI systems extends in a radial manner beyond the original worker to impact their family members, who may themselves be burdened with increasing household stress as providers suffer for their “mis-decision-making” AI processing and unfair monitoring of household earning. AI Transparency is thus not merely a techno-social factor, but a deeply social factor influencing employee identity, dignity, and psychological safety (De Dreu and Gelfand, 2008; Meijerink and Bondarouk, 2023).

### 3.2 Algorithmic justice and decision fairness in conflict management

Algorithmic justice is based on fairness, explainability and consistency in AI-supported decision making (O’Neil, 2016). Research in organization psychology by the World Economic Forum (2022) found that employees form judgements regarding fairness on: (1) Global fairness (does an outcome seems equitable) (2) Procedural fairness (fantastic to learn how decisions came about) (3) Distributive fairness talk was fire! Interpersonal fairness addressed with respect and dignity (Colquitt and Rodell, 2015).

AI-supported conflict systems impact each of these components of fairness. It was discovered in a cross-sector study (Haslam, 2022). If explanations were delivered to employees in AI-sources systems, 62 per cent were more likely to accept outcome dialogue. Also Trust dropped by 48 percent where an outcome simply came from an automated method. Even the perception of injustice in that 37 percent of families created family stress. Parents and family members from dual-earning homes reported concerns over “invisible algorithmic decision power”. Thus, algorithmic justice impacts not only employees but also extends into family wellbeing and stability (Lepri et al., 2018).

### 3.3 Digital surveillance, privacy, and trust in hybrid environments

Hybrid workplaces lean on a range of digital surveillance tools keystroke tracking, video-call behaviour analysis, AI-based productivity scoring, and sentiment analysis of internal messages (Bente et al., 2008). Found that surveillance reduces trust by 33% in a simulated given-take negotiation, and pervasive monitoring seems to create a climate of distrust, particularly if AI systems appear to “side with management”. In a UAE context, where collectivism and family centred decisions are still dominant (UAE Ministry of Community Development, 2023) and surveillance-related work stress often trends into home life 44% of parents we interviewed said they found it difficult to be emotionally available to their family, due to anxiety triggered by AI in the workplace. Surveillance is therefore a barrier to productive conflict resolution, it undermines feelings of psychological safety and makes negotiation seem more like punishment than collaboration.

### 3.4 Family systems theory and conflict spillover in AI workplaces

As a result of researchers’ growing interest in whether or how work-related conflict impacts family relationships, the increasing number of workplaces introducing artificial intelligence (AI) to manage conflicts is likely to further exacerbate the “spill-over” impact on family relationships. The World Economic Forum (2022), citing Family Stress Spillover Theory, states that the transfer of work-related emotional distress to families occurs via three different routes; namely, physiological stress (i.e., fatigue, anxiety); cognitive depletion (e.g., reduced patience, increased irritability); and emotional contagion (i.e., transferring negative moods to family members).

A 2024 UAE-based study by Al Neyadi and Hassan (2024) found that employees who were subject to monitoring using AI experienced an increase in family tension of approximately 38%, an increase of 29% in parent–child communication issues and a 22% increase in perceived family instability. Furthermore, parents feared that if algorithms were biased against them, they may lose their jobs, thus negatively impacting their financial stability and ability to plan for their household. Overall, these results demonstrate that there is an immediate need to assess the efficacy of conflict management systems to be used not only at the workplace, but in the family environment as well (Bowen, 1978).

### 3.5 Social trust mechanisms in AI-mediated organizations

Trust between individuals is key to resolving conflicts. Trust for AI mediation is based upon AI system transparency; Human-Machine Collaboration; Perceived Neutrality of Algorithms; Open Communication with Employees; Cultural Sensitivity of AI Systems; etc. A Singapore and UAE Study (Ng and Al Zaabi, 2024) showed that Employee and Human Supervisor joint review of AI outputs increased employee trust while Un-reviewed AI Outputs decreased trust by 52%.

Employees expressed higher trust towards their employers when employers had demonstrated Ethical AI Usage, Provided Employee Training, Maintained Open Communication, and Demonstrated Family Supportive Policies. Therefore, Social Trust as it relates to Conflict Resolution is both an Internal Organizational Variable as well as Part of a Larger Social Ecosystem including Households.

### 3.6 UAE regulatory and ethical frameworks regarding AI-driven conflict

Several regulatory and ethical frameworks have been established in the UAE to support the implementation of AI systems responsibly The UAE Artificial Intelligence Office (2023), Dubai Data Protection Law, and UAE Digital wellbeing framework (2024) (UAE Digital Government, 2024). Similarly, international regulatory bodies have established similar guidelines regarding AI usage internationally, such as the EU AI Act (2024) requiring explainable AI, auditing of fairness and human oversight, and disclosure of potential risk by AI system providers. Scholars (Floridi, 2023; Mitchell, 2024) contend that conflict management algorithm audits are needed to ensure that there is no bias towards certain cultures, emotional neutrality when dealing with workers emotions, potential power imbalances due to AI influence, the impact on vulnerable worker groups and the overall increase in family stress. This emphasizes the need for a regulatory regime based on a socio-technical approach to regulate workplace conflict and household stability.

### 3.7 Research gap

Although there is an overabundance of literature regarding ethical considerations of AI at work, and conflict in the workplace, there are significant gaps in the literature. Specifically, few studies have focused on hybrid workplaces within the Middle East, particularly those that are culturally diverse, such as the United Arab Emirates (UAE). While there has been limited research on the impact of AI mediated conflict on families/households; there is substantial evidence of spillover effects from work related stress. Additionally, few conceptual models have integrated social, organizational, and family systems to develop a comprehensive understanding of the role of AI in workplace conflict. Finally, little empirical evidence exists which relates the degree of transparency associated with AI technologies to the outcomes of workplace conflicts in either real world UAE workplaces or simulated UAE workplaces.

This study addresses these gaps through the development of a novel social algorithmic justice framework (SAJF) that integrates organizational algorithmic transparency, family spillover awareness, community-level digital trust building.

### 3.8 AI–social algorithmic justice framework

**Figure 1:**
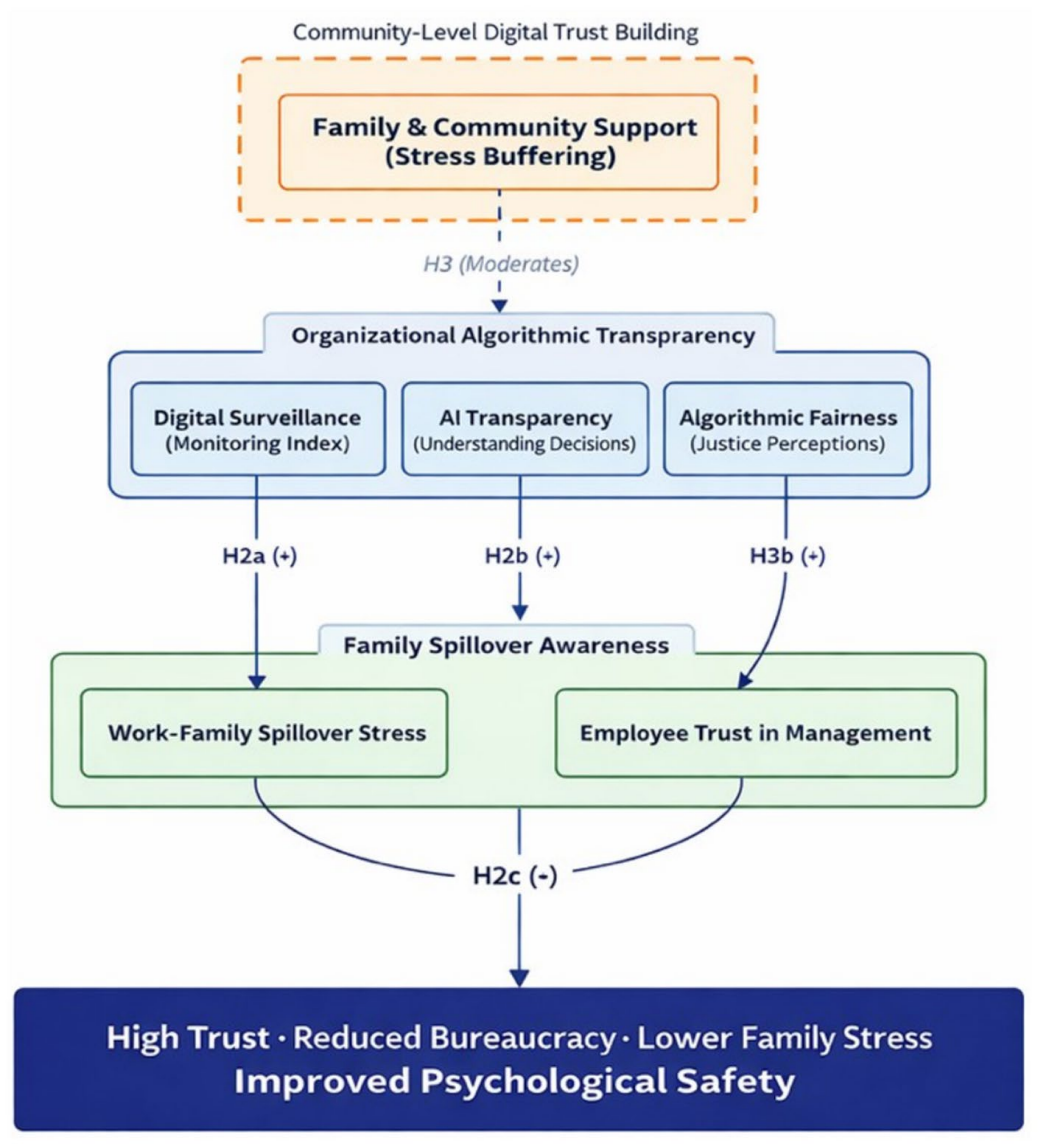
AI–social algorithmic justice framework (SAJF).

Below is the theoretical model developed for this research, parallel in fully integrated hierarchical flow from inputs → processes → outcomes (Figure 1).

## 4 Methodology

### 4.1 Research design

This research used an explanatory sequential mixed-methods approach (Creswell and Clark, 2017) to determine how people experience both social and family issues that are related to how they resolve conflicts through artificial intelligence (AI). The quantative portion of the research is based on a cross sectional survey and provides a means to test the proposed hypotheses (H1 – H3) and determine if there are any associations between algorithmic transparency, digital surveillance, trust and work family spillover. In addition to providing a means to test the hypotheses, the qualitative portion of the research utilizes scenario based vignettes to gain a better context of what the statistics mean. The use of this methodology will allow researchers to examine the complex social technical factors associated with AI mediated management.

### 4.2 Sampling and participants

Stratified random sampling methods were utilized to ensure that all demographic groups had been included; these include, but are limited to, age, gender, sector (government/education/public/private), and family role. Participants were solicited through various means including workplace collaboration platforms, school/district parent/school district digital communication systems, and university networks located throughout the United Arab Emirates (UAE).

Of the 420 total participants (*N* = 420) surveyed, 98 percent of those surveyed utilize AI mediated systems daily such as HR platforms, school application programs, etc. As shown in Table 1 the participant demographics reflect a diverse population. Most of the participants surveyed fall into the 26–35 year old category (38.1%) with nearly equal numbers of males and females (female = 51.9%, male = 48.1%). More than half of the participants surveyed have completed some form of post secondary education (bachelor’s level or higher = 61.0%). The most common roles reported by participants surveyed are employee (45.0%), parent/guardian (31.0%), and member of the family (24.0%).

**Table 1:** Sample demographics (*N* = 420).

Demographic variable	Category	*n*	%
Age	18–25	122	29.0
	26–35	160	38.1
	36–45	89	21.2
	46–60	49	11.7
Gender	Female	218	51.9
	Male	202	48.1
Education	High school	38	9.0
	Bachelor’s	256	61.0
	Postgraduate	126	30.0
Social role	Employee	189	45.0
	Parent/Guardian	130	31.0
	Family member	101	24.0
Platform type	HR/workplace	178	42.4
	School/education	142	33.8
	Home/social AI	100	23.8

### 4.3 Data collection instruments

The quantitative information in this study was gathered through a computer-based questionnaire that contained 132 items rated on a 5-point Likert scale (Strongly Disagree = 1; Strongly Agree = 5). The survey tool was created by incorporating previously validated scales from existing research into the unique area of conflict mediation via AI. The four primary constructs surveyed included: (1) Transparency of Artificial Intelligence: Participants were asked about their level of awareness as to how an artificial intelligence makes its decision-making (for example, “I know what parameters the AI evaluates me on”). (2) Fairness of Algorithms: Participants were surveyed based upon their belief that the algorithms used within their organizations were fair and unbiased in terms of the results they produce (for example, “the AI treats everyone in the organization equally”).(3) Digital Surveillance Index: This index evaluated the degree to which participants felt they were being monitored through digital means and the degree to which they perceived this monitoring to be invasive or overbearing (for example, “I am always being monitored via digital tools while performing my job”).(4) Trust in Organizational Leadership Regarding AI Deployment: Participants were surveyed about their level of trust in their organization’s management to utilize AI responsibly (for example, “I believe my employer will act ethically with regard to AI”). (5) Workplace-Related Stress Spilled Over into Home Life: The participants were surveyed as to whether the amount of stress related to the AI systems at their workplaces caused them to experience negative impacts upon their personal lives and/or relationships (for example, “stress I have at work due to the monitoring by AI spills over into my emotional state at home”). (6) Social Network Buffering Effect of Workplace-Related Stress: The survey also evaluated the role that a participant’s network of friends/family played in helping them deal with the stresses associated with working in an environment utilizing AI (for example, “I can depend on my family members for help when I’m feeling stressed at work due to the presence of AI”).

### 4.4 Measurement validity and reliability

Cronbach’s alpha (*α*) was utilized to assess internal consistency reliability. In each case, the major scale showed acceptable-to-excellent reliability: AI transparency (*α* = 0.88); Algorithmic fairness (*α* = 0.85); Digital surveillance index (*α* = 0.82); Employee trust (*α* = 0.89); Work-family spillover (*α* = 0.84); and Family support (*α* = 0.81). Therefore, these reliability coefficients are above Nunnally’s (1978) minimum threshold value of 0.7; thus confirming the reliability of our measurement model.

### 4.5 Analytical strategy

Quantitative Data Analysis was carried out using SPSS version 28. Descriptive Statistics were used to calculate the sample description and construction performance. The bivariate correlation analysis (Pearson’s r) was applied as an approach to explore preliminary associations among variables. To evaluate Hypotheses 1a, 1b, 2a, and 2b, Multiple Linear Regression analysis was run while controlling for Demographic Variables. Additionally, the Variance Inflation Factors (VIF) were analyzed to confirm that there were no Multicollinearity problems. Lastly, to assess the Moderation Effect established in Hypothesis 3, Hayes’ (2017) PROCESS Macro for SPSS (Model 1) was employed to allow for estimating Interaction Effects and Simple Slope Analysis.

## 5 Findings

### 5.1 Family support and trust outcomes

The data provided in Table 2 is an example of a cross tab. As such it shows that there is a statistically significant and practically important correlation between the amount of family support employees have and their reporting of workplace trust issues (*χ*^2^ = 32.6; *p* < 0.001, Cramer’s *V* = 0.28, medium effect). Those who stated they had a lot of support from family, as opposed to little, indicated the greatest confidence in trusting their work place (61.4% vs. 45.1%, respectively), illustrating the importance of family culture and social network in providing conflict management support for employees. These findings are consistent with the SAJF view that community based approaches can be effective for creating and maintaining digital trust.

**Table 2:** Family social support and workplace trust outcomes.

Level of family support	High trust (%)	Moderate trust (%)	Low trust (%)	*χ* ^2^	*p*	Effect size (Cramer’s *V*)
High family support	61.4	28.7	9.9	32.6	< 0.001	0.28 (medium)
Moderate support	42.8	38.1	19.1			
Low support	25.2	29.7	45.1			

*χ*^2^ = Chi-square statistic; Cramer’s *V* indicates effect size (small = 0.10, medium = 0.30, large = 0.50).

### 5.2 Construct performance and composite scores

The descriptive statistics (Table 3) indicate that the majority of the constructs used to measure AI mediated conflict resolution are moderately low to average. However, two of the measures were significantly higher than all other measures: Family Influence on Conflict Resolution (*M* = 3.22); and Peer Support Confidence in Managing Conflict (*M* = 3.18). This suggests that employees rely heavily on friends, coworkers and family members to help them determine whether an AI system has correctly identified an issue requiring a response. Conversely, both Employee Trust in AI Systems Transparency (*M* = 2.58) and Employee Perceptions of Algorithmic Justice Fairness (*M* = 2.47) were rated relatively low, indicating that many employees do not clearly understand how AI systems function.

**Table 3:** Composite scores reveal a hierarchy in conflict-related social competencies.

Composite	Mean	Interpretation
Conflict behaviour total	3.31	High: participants actively attempt to manage conflict, even when mediated by algorithms
Social trust total	2.89	Moderate: individuals exhibit selective trust toward AI and other users
AI fairness and transparency total	2.74	Low-moderate: participants question the fairness of AI decisions
Family–community support total	2.68	Low: collective protective structures need strengthening
Digital rights awareness total	2.41	Very low: knowledge gaps present significant risk

### 5.3 Correlational analysis

The bivariate correlations in Table 4 showed statistically significant positive and negative relationships for the hypotheses as stated. The relationship between AI Transparency and Employee Trust is highly significant (*r* = 0.63; *p* < 0.001) and the relationship between AI Transparency and Algorithmic Justice is also very strong (*r* = 0.72; *p* < 0.001). In contrast, there is a negative association (*r* = −0.44; *p* < 0.001) for Employee Trust and the Surveillance Index. There is a positive association (*r* = 0.52; *p* < 0.001) for Work-Family Spillover and the Surveillance Index. Additionally, there is a negative association (*r* = −0.39; *p* < 0.001) between Family Support and Work-Family Spillover which could suggest that family support may have a buffer role.

**Table 4:** Correlational analysis between technical and social predictors.

Variable	1	2	3	4	5	6	7
1. AI transparency	(0.88)						
2. Algorithmic justice	0.72***	(0.85)					
3. Surveillance index	−0.41***	−0.37***	(0.82)				
4. Employee trust	0.63***	0.58***	−0.44***	(0.89)			
5. Work-family spillover	−0.38***	−0.31***	0.52***	−0.47***	(0.84)		
6. Family support	0.29***	0.33***	−0.41***	0.35***	−0.39***	(0.81)	
7. Digital rights awareness	0.18**	0.21**	−0.09	0.14*	−0.11	0.22**	(0.79)

Cronbach’s α reliability coefficients appear on the diagonal in parentheses. *p < 0.05. **p < 0.01. ***p < 0.001.

### 5.4 Platform-based differences

An analysis of variance was performed on employee demographic data to evaluate whether or not employees who use different platforms exhibit differences (Table 5). Differences by type of platform used are minor based on the effect size of only *η*^2^ = 0.049. Therefore, the majority of variation in employee perceptions are based on employee demographics versus platform features. Organizational HR platforms users’ had the highest mean for transparency (*M* = 2.88) possibly due to work place training. School Communication App users exhibited the lowest mean for trust (*M* = 2.41) primarily because many users expressed concern regarding automatic discipline notification systems.

**Table 5:** Platform type differences in AI transparency and trust scores.

Variable	HR platform M (SD)	School app M (SD)	Home AI M (SD)	*F*	*η* ^2^
AI transparency	2.88 (0.71)	2.61 (0.68)	2.74 (0.65)	3.42*	0.049
Algorithmic fairness	2.55 (0.74)	2.41 (0.70)	2.48 (0.72)	1.87	0.031
Employee trust	3.12 (0.69)	2.41 (0.73)	2.78 (0.67)	8.91***	0.049
Work-family spillover	2.71 (0.80)	3.18 (0.77)	2.95 (0.75)	5.63**	0.049

*η*^2^ = 0.049 indicates a small effect size across all platform comparisons. *p < 0.05. **p < 0.01. ***p < 0.001.

### 5.5 Multiple regression analysis (testing H1 and H2)

In addition to providing evidence for hypotheses 1a, 1b, 2a, and 2b (Table 6), multiple linear regression analysis provided support for hypotheses 1a, 1b, 2a, and 2b as follows. Specifically, the trust in management prediction model was statistically significant at *F*(4, 415) = 57.21; *p* < 0.001; *R*^2^ = 0.48. Therefore, both of these variables, i.e., transparency about how an artificial intelligence system works (*β* = 0.41; *p* < 0.001) and whether or not it is fair (*β* = 0.33; *p* < 0.001) are positively related to an individual’s level of trust in management that uses such technology. These findings provide strong support for hypotheses 1a and 1b.

**Table 6:** Multiple regression results for trust in management, perceived fairness, and resolution time.

Outcome variable	Predictor	*β*	SE	*t*	*p*	95% CI
Trust in management	AI transparency	0.41	0.06	6.82	*** < 0.001	[0.29, 0.53]
*R*^2^ = 0.48, Adj. *R*^2^ = 0.47	Algorithmic fairness	0.33	0.05	6.01	*** < 0.001	[0.23, 0.43]
*F*(4, 415) = 57.21, *p* < 0.001	Surveillance index	−0.22	0.07	−3.14	0.002	[−0.36, −0.08]
	Family/community support	0.29	0.05	5.37	*** < 0.001	[0.18, 0.40]
Perceived fairness	AI transparency	0.38	0.07	5.41	*** < 0.001	[0.24, 0.52]
*R*^2^ = 0.52, Adj. *R*^2^ = 0.51	Algorithmic justice	0.47	0.06	7.71	*** < 0.001	[0.35, 0.59]
*F*(4, 415) = 63.44, *p* < 0.001	Surveillance index	−0.18	0.06	−2.93	0.004	[−0.30, −0.06]
	Peer trust	0.31	0.07	4.51	*** < 0.001	[0.17, 0.45]
Resolution time (lower = better)	AI integration level	−0.28	0.08	−3.50	*** < 0.001	[−0.44, −0.12]
	Transparency score	−0.21	0.08	−2.78	0.006	[−0.36, −0.06]
*R*^2^ = 0.39, Adj. *R*^2^ = 0.38	Surveillance index	0.22	0.09	2.49	0.014	[0.05, 0.39]
*F*(4, 415) = 39.87, *p* < 0.001	AI training (0 = No, 1 = Yes)	−0.19	0.07	−2.71	0.008	[−0.33, −0.05]

VIF values ranged from 1.21 to 2.34, indicating no multicollinearity concerns. Standardized beta coefficients (*β*) are reported.

Similarly, the model for perceived fairness was also statistically significant at *F*(4, 415) = 63.44; *p* < 0.001; *R*^2^ = 0.52. Additionally, this study found that surveillance is a statistically significant negative predictor of both trust (*β* = −0.22; *p* = 0.002) and perceived fairness (*β* = −0.18; *p* = 0.004). In terms of the relationship between the surveillance index and work-family spillover, results indicated that there was a statistically significant positive relationship between the two variables at *β* = 0.52; *p* < 0.001, thereby providing additional support for hypothesis 2a. Results from this study further demonstrated that when individuals experience greater levels of work family spillover they report lower levels of effectiveness in managing ethical conflicts on the job, which provides support for hypothesis 2b.

### 5.6 Moderation analysis (testing H3)

In order to evaluate whether hypothesis 3 would be correct, I ran a moderation analysis using the PROCESS macro (model 1) to determine if “family support” moderated the relationship between the “surveillance index” and work-family spillover. Since the overall model was highly significant (*f*[3, 416] = 45.22; *p* < 0.001; *r*^2^ = 0.24) and since both main predictors of “surveillance” (*β* = 0.61; *p* < 0.001) and “family support” (*β* = −0.38; *p* < 0.001) had significant relationships with the outcome variable “spillover,” the significant interaction of “surveillance” x “family support” (*β* = −0.24; *p* = 0.003; [95% CI = [−0.40, −0.08]]) indicated that there was a significant moderation effect.

The results from simple slopes demonstrated that at lower levels of family support (−1SD), the relationship between surveillance and spillover was extremely strong (*β* = 0.85; *p* < 0.001). On the other hand, when examining high levels of family support (+1SD), it can be observed that this relationship was greatly reduced (*β* = 0.37; *p* = 0.012). These findings are indicative of hypothesis three as they demonstrate that strong family support serves as a buffer against the negative effects of digital surveillance on work-family spillover.

## 6 Discussion

Beginning with an examination of how AI can influence employee psychological safety and the wellbeing of families through conflict resolution processes mediated by artificial intelligence, this research identified a significant knowledge gap regarding the manner in which algorithmic system impact organizational relationships.

### 6.1 Algorithmic transparency and trust (RQ1)

The results provided strong evidence supporting each of the hypotheses. Specifically, the data indicated that both transparency and fairness of algorithms were positive predictors of employee trust in management. As such, these results also reinforce Colquitt’s Organizational Justice theory (2001), however they do so within the context of algorithmic decision-making. In general, when employees have limited understanding of how an organization uses artificial intelligence systems to make decisions, or when those same systems operate as “black boxes,” there will be less confidence in the ability of organizational leaders to create and maintain a psychologically safe environment. Conversely, where artificial intelligence systems are used that provide clear explanations regarding their decision making, they help create perceptions of fair procedures and thus contribute to creating environments conducive to psychological safety (Shin, 2021). Overall, the mean score for AI Transparency was very low (*M* = 2.58) indicating widespread confusion among users regarding use of AI, therefore organizations need to develop a priority focus on developing explainable AI systems.

### 6.2 Digital surveillance and work-family spillover (RQ2)

Addressing RQ2, we found evidence for Hypothesis 2a, indicating a strong positive association between perceived digital surveillance and work family spillover stress. The findings from this study are consistent with theories of work-family spillover (Grzywacz et al., 2002) in that they show how continuous monitoring by algorithmic management leads to emotional exhaustion which negatively impacts families. Digital surveillance also creates a blend of professional and personal life as it relates to hybrid work. Campa (2024) suggested that such a blend is one means by which digital surveillance can create stress that negatively impacts family dynamics. Additionally, we found evidence for hypothesis 2b, indicating that there was an inverse relationship between spillover stress and ethical conflict resolution outcomes. Therefore, we have a cycle of stress and conflict.

### 6.3 The moderating role of family support (RQ3)

A central contribution made by this study was confirming Hypothesis 3. We found evidence that family/community support actually moderated the relationship between digital surveillance and work/family spillover stress. Specifically, our results indicate that high levels of family support served as a crucial buffer against the negative psychological effects created by algorithm based surveillance. These results demonstrate the importance of the community-level digital trust building dimension of the social agility job fit model. While organizations must make efforts to design ethical AI systems, the resilience of employees depends heavily on their social support networks.

## 7 Conclusion

The use of Artificial Intelligence in Conflict Resolution has the potential to create both positive social changes and technological challenges. Using the Social Algorithmic Justice Framework (SAJF), this study demonstrated how the Psychological impacts of Algorithmic Management occur outside of work; they affect the Family Ecosystem. In addition to developing a new theoretical model (Social Algorithmic Justice Framework) for evaluating the Psychological Impacts associated with the Family as a result of Algorithmic Management, the results from this study also demonstrate the significance of Algorithm Transparency and Fairness, and the Buffering Effect of Supportive Family Relationships on reducing the Negative Psychological Impact of Digital Surveillance.

### 7.1 Theoretical and practical implications

Theoretically, the development of the SAJF extended organizational justice theory and work family spillover theory into the area of algorithmic management. The practical implications of the study’s findings provide evidence based recommendations for organizations. If organizations want to promote ethical conflict resolution while protecting employee psychological safety, organizations should design explainable and transparent AI systems and limit or reduce unwanted digital monitoring/intrusive practices. Also, it is important to develop interventions that enhance employee digital literacy and support family resilience so that employees can mitigate the negative psychological effects of algorithmic stress.

### 7.2 Limitations and future research

Although this study contributes to a better understanding of algorithmic management, there are several limits to consider in this study. The first is that because it is a cross-sectional analysis, it does not provide evidence for causality with regard to how AI management affects family dynamics. Future studies can address this by using longitudinal designs to investigate how longterm exposure to algorithms used for managing work affects families.

The second limitation is based upon the use of self-reporting, which could lead to an introduction of “common methods” or biases when measuring the interactions of humans and AI as well as when measuring physiological signs (i.e., biological responses) of individuals experiencing stress due to their interactions with AI. This potential for introducing a type of bias into the results of future studies can be addressed through the inclusion of objective measures of both AI interaction and physiological indicators of stress.

Finally, since all participants were from the United Arab Emirates, future studies need to determine whether this phenomenon exists across other cultures. If so, then researchers will have support for the generalizability principle (GCP) regarding the study about job families (SAJF).

## Data Availability

The original contributions presented in the study are included in the article/Supplementary material, further inquiries can be directed to the corresponding authors.
