# Supplementary material for: Social and familial implications of algorithmic conflict management in hybrid work environments “a study of AI-driven fairness and trust in UAE organizations”
Source: Front Sociol. 2026 Jun 16;11:1754294. doi: 10.3389/fsoc.2026.1754294 (PMC13314403; doi:10.3389/fsoc.2026.1754294)
Supplement: Supplementary file 1 [file Supplementary_file_1.DOCX]

## **Supplementary Figures**


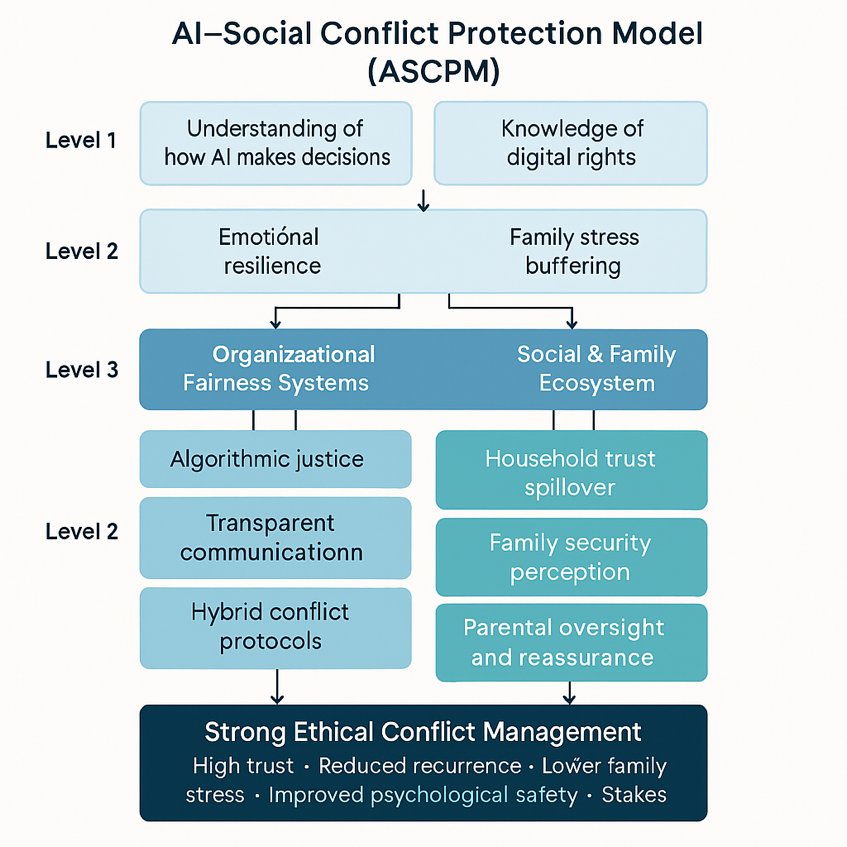


*Figure 1. AI–Social Conflict Protection Model (ASCPM)*


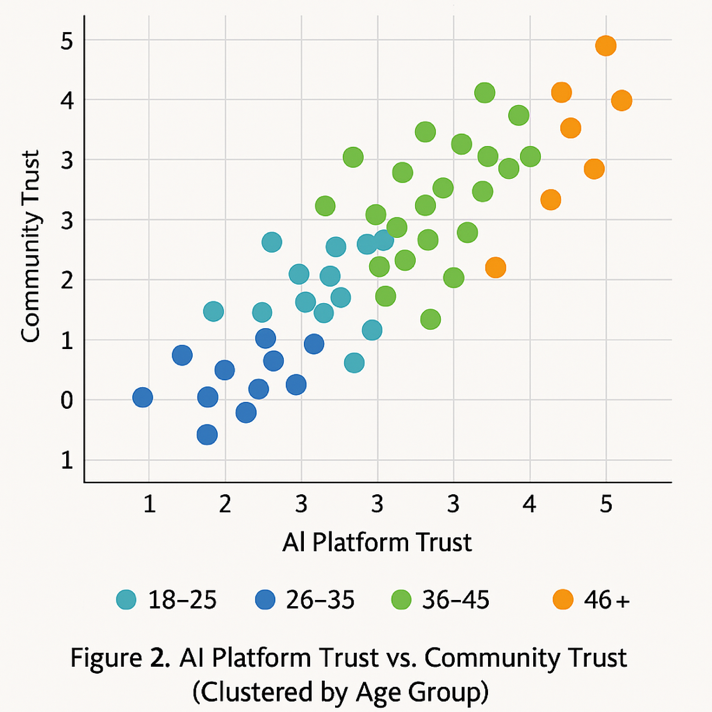


Figure2. AI PlatformTrust vs.Community Trust (clustered byAge Group)
